# Fish Husbandry Practices and Water Quality in Central Kenya: Potential Risk Factors for Fish Mortality and Infectious Diseases

**DOI:** 10.1155/2020/6839354

**Published:** 2020-03-19

**Authors:** Daniel W. Wanja, Paul G. Mbuthia, Robert M. Waruiru, Janet M. Mwadime, Lilly C. Bebora, Philip N. Nyaga, Helena A. Ngowi

**Affiliations:** ^1^University of Nairobi, College of Agriculture and Veterinary Sciences, Department of Veterinary Pathology, Microbiology and Parasitology, P.O. Box 29053-00625, Kangemi, Nairobi, Kenya; ^2^Sokoine University of Agriculture, College of Veterinary and Medical Sciences, P.O. Box 3000, Chuo Kikuu, Morogoro, Tanzania

## Abstract

Fish mortality has an enormous impact on the aquaculture industry by reducing fish production and slowing industrial growth. A cross-sectional study was carried out in Kirinyaga County, Central Kenya, to evaluate potential risks of fish mortality and disease transmission and suitability of pond water for rearing fish. A semistructured questionnaire that focused on general information, management practices, and disease history was administered to 92 small-scale fish farmers. Parasitological examination of fish sampled from selected farms (farms that were reporting mortality at the time of sampling) was done by following the standard procedure. Water quality parameters for 33 ponds were evaluated *in situ* (recorded on pond site) and *ex situ* (analysed at the laboratory) following the standard methods. The risks were assessed by adjusted odds ratio based on univariate regression analysis. Prevalent fish husbandry practices that were found to be associated with fish mortality and acquisition of pathogens in the study area were the use of raw livestock manure (0R = 1.500), high fish stocking density (0R = 1.168), and feeding fish on homemade rations (0R = 1.128). Parasitological investigation found infestation with *Diplostomum* spp., *Dactylogyrus* spp., *Clinostomum* spp., and *Piscicola* leeches. Water temperature and pH were found fit for rearing fish. Of the 33 fishpond water samples tested, 1 (3%) and 6 (18%) exceeded the recommended limits of <100 mg/L and <0.2 mg/L of nitrate and nitrite, respectively. Of the 29 fishpond water tested, 15 (59%) exceeded the recommended limits of <100 mg/L of total ammonia. The findings show that the use of raw livestock manure, high fish stocking density, high nitrates and nitrites, and high ammonia levels in fishponds are potential risk factors for fish mortality and acquisition of infectious pathogens in a pond environment in a rural setup, in Central Kenya. There is a need to address the above factors in small-scale farming practices to minimize fish loss and also to prevent the occurrence and spread of infectious pathogens.

## 1. Introduction

Aquaculture plays an important role in provision of food, livelihood, and source of income in third world countries including Kenya. Growth of the aquaculture sector is occasioned by adoption of novel scientifically proven practices and rising demands for fish aquatic protein [1]. Due to expansion of the sector, there is a rapid shift from extensive methods to semi-intensive and intensive culture methods with the potential of producing higher output or turnover. It is prudent to justify that their main and intended role is to optimize fish production; however, intensification in aquaculture comes with risks of diseases. Fish farmers are experiencing massive losses, situation that discourages new farmers to venture in the sector. While diseases have been documented as the most limiting constraints, a good number of disease-causing agents are ubiquitous in water and cause few problems; however, an imbalance due to water quality fluctuations and husbandry practices results in disease occurrence.

According to Huicab-Pech et al. [2], a higher percentage of fish diseases (90%) in a controlled aquatic environment are associated with improper husbandry practices and inadequate implementation of biosecurity measures. Healthy fish having latent infections usually do not succumb to such infections as long as favourable culture conditions continue to prevail [3]. However, external stressors such as poor water quality, high stocking density, transportation stress, and improper nutrition may exacerbate the situation to a clinical disease, sometimes often fatal. Moreover, lack of farmer knowledge regarding the absence or presence of pathogenic risks, low cost of food production, and intensification of pisciculture systems further worsens the issues, as discussed by Huicab-Pech et al. [2]. Aquaculture systems are discernibly different as they relate to aspects of parameters of control. Excluding water source from the equation, water quality is affected by the management practices among other things. As a way of enhancing market value and fish survival, several essential husbandry strategies ought to be regularly engaged.

Water quality is the most important limiting factor in rearing of fish and directly affects feed efficiency, growth rate, the fish's health, and survival [4]. Aquatic life is highly dependent on physical, chemical, and biological factors of water, playing a substantial role in the biology and physiology of fish [5]. For fish to survive within its desired limit, fluctuations within the ranges must be gradual because a rapid change in the water quality parameters will seriously stress and reduce resistance to diseases and sometimes be fatal to fish. Different fish species have different tolerance limits for water quality parameters, within which they can survive, grow, and reproduce. Increasing incidence of fish deaths attributed to poor water quality has forced many farmers to abandon aquaculture [6]. It is therefore important to monitor water quality parameters to ensure they remain within the optimum range of cultured fish.

To establish gaps for extension services that could support the sustainability and expansion of aquaculture industry in Kenya, basic information about farming practices is needed. Inadequate fish farmers' management practices may have negative implications on fish survival. The aim of this study was to investigate fish farming practices in small-scale fish farms in Kirinyaga County that may result in occurrence of fish pathogen and disease transmission and affect fish survival and the suitability of pond water for fish farming.

## 2. Materials and Methods

### 2.1. Ethics Statement

The study protocol was approved by the Biosafety, Animal Use and Ethics Committee of the Faculty of Veterinary Medicine, University of Nairobi, Kenya. Informed consent was obtained verbally from fish farmers to participate in the study.

### 2.2. Study Area

This study was conducted in Kirinyaga County, Central Kenya, which is located between latitudes 0°1′ and 0°40′ South and longitudes 37° and 38° East at an altitude of 1230 m. Administratively, the County has five subcounties: Kirinyaga Central, Kirinyaga West, Kirinyaga East, Mwea East, and Mwea West. The county has a tropical savannah climate and an equatorial rainfall pattern. The county was chosen as it has great potential for fish farming due to favourable resources and climatic/hydrological conditions [7].

### 2.3. Study Design

A cross-sectional survey was carried out to evaluate fish husbandry practices and fish health history, during December 2017 and May 2018. Data were collected through semistructured questionnaire interviews to available and willing respondents who had to be either the owners of the fishponds or took part in the management of the same. The questionnaire focused on background information (fish species and culture type, water sources, and pond type), management practices (pond fertilization type, stocking density, feed sources, and seining practices), disease history and biosecurity practices, and health management.

The interviews were supplemented with on-farm veterinary visits to observe the farming sites and operations as well as the surrounding environment. The questionnaire was initially pretested in the study area for clarity and time management. After pretesting, they were revised and corrected accordingly and then a final set of questionnaire was made.

In addition, physicochemical parameters (dissolved oxygen water temperature and pH were recorded *in situ* while total ammonia, nitrate, and nitrite were analysed in the laboratory) of selected ponds were assessed for their suitability for fish production.

### 2.4. Sampling Strategy and Sample Size Determination

Households were the sampling units used in the study. A list of fish farmers in different subcounties was provided by respective subcounty fisheries extension officers. The list of fish farmers formed the sampling frame. A purposive sampling was used to obtain active and available fish farmers and participant fish farmers who were drawn from the population of fish farmers in all subcounties of Kirinyaga County.

The minimum sample size was guided by a formula given by Yamane [8]: *n*=*N*/(1+*N*(*e*)^2^) where *n* is the sample size, *N* is the population size (290 active farmers), and *e* is the level of precision (0.05). This gave a sample size of 168. However, only 92 farmers were selected randomly with the help of the subcounty fisheries extension officers from all the 5 subcounties of Kirinyaga County as follows: Kirinyaga West (19), Mwea East (12), Kirinyaga Central (20), Mwea West (14), and Kirinyaga East (27). The number of farmers selected to participate in the study was guided by the number of fish farmers in the subcounty and the existing resources available for the study.

### 2.5. Necropsy and Parasitological Examination

Fish sampling was done in farms that were reporting incidences of mortality at the time of the study. Consent to sample was sought from fish farm owners. Ten (10) fish were obtained from each of the 23 farms that had incidences of fish mortality. Necropsy procedure was undertaken as described by Noga [9]. Fresh skin scrapping/slime, gills scrapes, and eyes contents were examined microscopically on-site.

### 2.6. Measurement of Water Quality and Water Sampling for Chemical Analysis

To assess water quality as a predisposing risk factor influencing disease transmission, selected physicochemical parameters of water in selected ponds were measured early in the morning (0800 hrs) on the day of questionnaire interviewing in the respective farms.

Water temperature and dissolved oxygen were measured *in situ* using handheld Hanna Multiprobe meter (model Hi-9142, Hanna Instruments Inc., USA) while pH was measured *in situ* using handheld Hanna meter (model Hi-98127, Hanna Instruments Inc., USA). Ammonia concentration was measured using a portable Hanna ammonia medium-range meter (model Hi-96715C, Hanna Instruments Inc., USA). Water sampling for further chemical analysis was done by following the standard methods for examination of water and wastewater developed by the American Public Health Association (APHA) [10].

Water samples were collected in 2 L sterile containers. The samples were then transported to the laboratory where they were frozen before submission to Government Chemist laboratories for analysis. Total nitrite and nitrate were analysed, in the laboratory using a HACH DR/2010 Spectrophotometer following the standard methods by APHA [10].

### 2.7. Data Analysis

The collected data were validated, coded, entered, and stored in Microsoft Excel® and then exported onto Statistical Package for the Social Sciences (SPSS®), version 22.0 for analysis. Logistic regression analysis with estimation of odds ratios (OR) was used to examine the significance of associations of risk factors with the outcome variable. Chi-square (*χ*^2^) was used as a measure of significance, while Cramer's V was used to measure the strength of association.

## 3. Results and Discussion

### 3.1. General Information

#### 3.1.1. Fish Species Kept and Culture Type

Nile tilapia (*Oreochromis niloticus*) was the most cultured species, followed by African catfish (*Clarias gariepinus*), ornamental (koi carp (*Cyprinus carpio carpio*) and goldfish (*Carassius auratus*)), and lastly rainbow trout (*Oncorhynchus mykiss*). Tilapia was the dominant species kept by the farmers under either mixed-sex or monosex monoculture system in the study area. Monoculture was more popular with tilapia at 51% (73/144) than catfish (31%). The commonest farming system practiced in Kirinyaga County was tilapia mixed-sex monoculture (32%). Tilapia-catfish polyculture was less common, practiced by only 10% (14/144) of fish farmers.

A cross-sectional study conducted by Jacobi [11] in Western Kenya reported that most farmers (74%) cultured Nile tilapia and African catfish in monoculture systems, while catfish-tilapia polyculture was practiced by 26% of the farmers. The polyculture in Western was higher than in Kirinyaga in Central Kenya. Low uptake of polyculture has been attributed to poor knowledge among fish farmers [12, 13].

**Figure 1:**
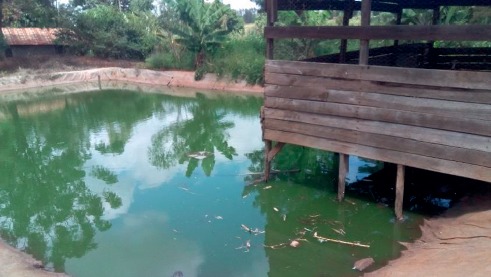
A liner pond stocked with fish integrated with poultry (chicken) farming.

Fish farming activities can be integrated with livestock, e.g., goats, rabbit, cattle, and chicken (Figure 1) and crops (rice) production. This is, however, a less popular farming technology in Kirinyaga County, practiced by 21% of the fish farmers.

Despite enormous potential of integrated farming technology in conserving resources while snow-balling farm earnings, it remains a less popular activity in the county. Lack of documented information showing the productivity potential and a lot of technical information have been reported as the possible hindrance [14]. Challenges facing integrated livestock-fish aquaculture are government laxity on the aquaculture sector, weak aquaculture research and applications, and political interferences, as well as cultural challenges [14].

#### 3.1.2. Type of Pond

**Figure 2:**
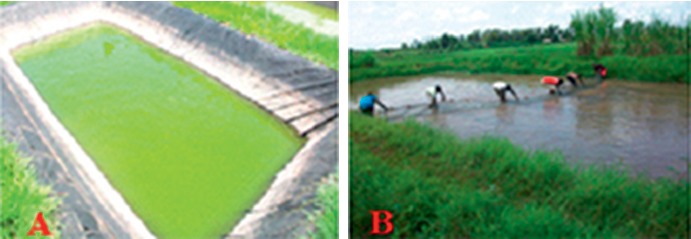
A plastic liner pond (a) and an earthen pond (b) with fish harvesters in Kirinyaga.

Most fishponds (68%; 63/92) were ultraviolet-treated plastic-lined ponds (Figure 2(a)) followed by earthen ponds at 25% (23/92) (Figure 2(b)) and the rest were concrete ponds. Many fishponds were situated in midland and highland areas of Kirinyaga County where the soil has a low content of clay soil [15]. Plastic liners are ideal in such areas so as to prevent seepage [16].

#### 3.1.3. Pond Water Supply and Frequency of Changing Pond Water

The main sources of water used for fish farming were permanent rivers at 61% (56/92), followed by public piped untreated water at 27% (25/92). Kirinyaga County has six major rivers with at least one river flowing through each subcounty. The rivers are Sagana, Nyamindi, Rupingazi, Thiba, Rwamuthambi, and Ragati, all of which drain into the Tana River [7].

Nearly all the farmers did not sterilize water before use. Majority (31%, 29/92) of farmers refilled/topped up their pond when water decreased below a certain point, while 24% of the farmers neither changed nor refilled water in their fishponds during a production cycle.

### 3.2. Fish Farming Practices

#### 3.2.1. Pond Preparation and Treatment

Many fishponds (49%, 45/92) were drained and the bottom sediment/mud removed after harvesting. Some ponds (23%) had never been harvested since they were stocked, 16% did not drain their ponds, and 12% only drained their fishponds without removing bottom mud. Majority of farmers (70%, 64/92) did not treat their fishponds, 14% treated their ponds by liming and 13% by natural dry out, while 3% relied on combination of both liming and natural dry out as pond treatment techniques before restocking fish.

Pond bottom treatments between crops aim at oxidizing wastes; eradicating predators, pathogens, and vectors of pathogens; improving the soil pH; increasing availability of planktons before restocking; and removing or redistributing sediment [17]. This was not well done in the study area, increasing the chances of exposing new fish stocks to infectious pathogens from previous fish stocks.

#### 3.2.2. Type of Fertilizer Used

Most fish farmers in Kirinyaga County fertilized their fishponds (82%, 75/92) while 18% did not fertilize their fishponds. A high proportion of farmers (48%) used raw organic fertilizers (livestock manure) compared to 12% who used inorganic/chemical fertilizers. Twenty-two percent reported to have used both organic and inorganic fertilizers. For livestock manure, dung of cow, poultry goat, and pig were commonly used while diammonium phosphate (DAP), calcium ammonium nitrate (CAN), and urea topped list of chemical fertilizers used. The use of these fertilizers has been reported elsewhere by Jackson [18], who also reported using nitrogen: phosphorous: potassium (NPK).

Most farmers practiced mixed farming, raising farm animals on or near the pond making it feasible for direct pond application of manure. In addition, animal manure is inexpensive compared to chemical fertilizers, cutting down the cost of production. Moreover, use of livestock manure in ponds increases the natural food supply to fish so as to minimize feed-related production costs [19].

#### 3.2.3. Fish Pond Stocking Density

Majority of small-scale farmers in the study area own a minimum of one pond to a maximum of 40 fishponds. The size of a pond in Kirinyaga County is variable, ranging from majority of 300 m^2^ to 15 m^2^. Many fish farms reared mixed-sex tilapia, monoculture catfish, catfish-tilapia polyculture, and koi carp-goldfish polyculture and had their ponds stocked with the recommended 3-4 fish per square meters. However, there were also incidences of either understocking or overstocking under different culture systems.

A stocking capacity of 3 fish per m^2^ is usually used in ponds in Kenya to generate 1 kg fish per m^2^ [20]. Munasinghe [21] reported that some shrimp farmers in Puttallam were shifting to lower density to minimize feed and labour costs. However, this explains the phenomenon in Kirinyaga County; losses from fish deaths have also been castigated as cause of lower densities. While high stocking density is taunted for high yield per unit volume of water under intensive system, most operations in Kirinyaga County are extensive to semi-intensive and therefore have no economic gains from high density. Moreover, overstocking under extensive systems leads to accumulation of fish faecal material which lead to poor water quality through the build-up of ammonia [22], as observed in this study.

#### 3.2.4. Feed Sources

Sixty-five percent (65%, 60/92) of the farmers interviewed used commercial feeds, 22% used homemade ration (livestock feeds/weeds), and 6.5% fed fish on both types of feed; however, 6.5% of the fish farmers did not feed fish at all. There was no difference in feeding regime to postfingerlings, grow-out, and mature fish. Similar work by Kapanda et al. [23] in Mchinji rural region, Malawi, reported inadequate knowledge on appropriate feeding among most farmers.

The quality of commercial fish feeds used in Kenya is variable with 24–30% and 30–40% crude protein for Nile tilapia and African catfish, respectively [24]. Commercial feeds are highly priced, and for this reason, most farmers end up constituting their own feed rations locally [25]. Unavailability of commercial feeds in the study area was another concern among farmers with good purchasing power. Feed materials and ingredients used locally and commonly in Kirinyaga County were livestock feeds (rice/wheat and/or maize bran) and plants (cassava leaves and sweet potato vines) and weeds. Liti et al. [24] and Munguti et al. [26] reported a similar trend across the country. Homemade rations are imbalanced diets (low protein content) that do not meet the nutritional requirement of fish leading to stunted growth and metabolic deficiencies [22].

#### 3.2.5. Seining Practices

Most farmers (63%, 58/92) shared fishing nets with other farmers, 29% did not share nets while 8% had not harvested the fish. A high proportion of farmers (71%, 65/92) used the same fishing net between own ponds, while 29% used a different fishing net for each pond. These nets are not disinfected between fish harvesting.

Sharing fishing gears and use of the same fish harvesters are a common practice in the aquaculture industry. Poverty or low purchasing power is to blame for this norm, as most farmers reported that they could not afford to buy own fishing gears. Fishing gears especially the seines may act as vehicles for transfer of infectious agents from one farm to another or one pond to another. Bebak et al. [27] in Alabama, USA, reported correlation between seining activities involving commercial fish harvesters with *Aeromonas hydrophila* transmission and/or outbreaks in cultured catfish. It is recommended to have separate equipment for use in different fishponds and prior disinfection.

### 3.3. On-Farm Observations

#### 3.3.1. Occurrence of Floods into Fishponds

**Figure 3:**
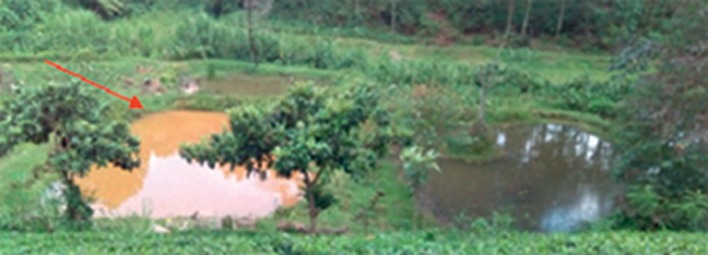
Flooded earthen pond (red arrow) next to none flooded pond. The ponds are constructed close to a river.

A significant number (15%) of farmers indicated to have experienced floods in their ponds. Floods were mainly reported in earthen ponds built on a valley or on a wetland (Figure 3). Some of the problems mostly reported as a result of floods included escape of fish from ponds, introduction of predators, death of fish, and disease outbreaks.

The impact of floods and related water quality stress can contribute to fish disease [28]. An instant change in water quality parameters during flooding causes fish stress and makes fish more susceptible to infectious pathogen including bacteria [29]. During floods, there is an inflow of silt and debris-laden water into the pond causing siltation, asphyxiation, and mass kills of the young fish [30].

#### 3.3.2. Excess Vegetation within and around the Pond and Abandoned Ponds

**Figure 4:**
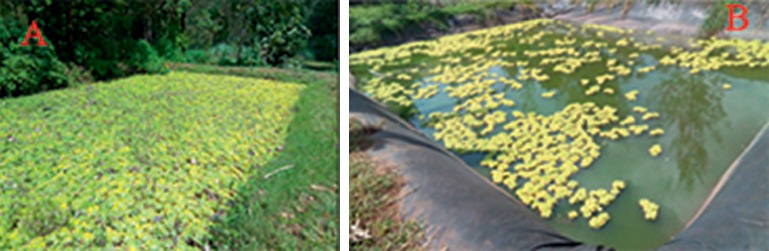
Photographs showing an earthen pond (a) and liner pond (b) with overgrown vegetation.

An observational analysis during farm visits showed that 10% of the fishponds in the study area were poorly managed with overgrown and excess vegetation (Figure 4).

Excess pond vegetation affects the aquatic environment by acting as a wind barrier, thereby causing depletion of dissolved oxygen and reducing sunlight penetration. Moreover, excess vegetation also acts as niches for fish parasites and pathogens as well as hiding places for fish predators: snakes, fowls, and crocodiles [30].

#### 3.3.3. Presence of Piscivourous Birds

The birds would be seen resting on trees near the pond. Birds litter on dykes of the ponds has been confirmed to play a possible role in the transfer of pathogens into aquatic life. Murugami et al. [31] in Kirinyaga County reported that piscivourous birds are involved in the transmission of fish parasites, e.g., digenean parasites. The water birds may also be involved in the transmission of bacterial agents, e.g., *Francisella* spp. and *Edwardsiella tarda* [32, 33] and viral pathogens. Moreover, water birds prey on fish, thus reducing aquaculture productivity and profitability [34].

### 3.4. Fish Disease History and Biosecurity Practices

#### 3.4.1. Fish Disease

In the areas of the county where questionnaires were administered, only 25% of the respondents rated fish diseases as a challenge in their farms; however, no farmer specified the name of fish disease. Most fish farmers (57%, *n* = 52) did not know how to identify sick fish on their culture systems. Most fish farmers interviewed (75%, *n* = 69) had never seen sick fish in their farms; however, 25% of the respondents reported sick/diseased fish with incidences of mortality in the last 6 months.

Aquaculture in Kenya is largely extensive to semi-intensive small-scale operations. Smallholder farmers are poverty-ridden and have inadequate knowledge on fish diseases [35]. Low disease reporting is attributed to lack of cognisance about fish diseases among farmers. However, most infectious diseases in fish are opportunistic [36]; stress can lead to fish mortalities. Stress may be due to mishandling, overcrowding, transportation under poor conditions, poor nutrition plane, and poor water quality. Xu et al. [37] experimentally demonstrated that *Ichthyophthirius multifiliis* enhanced *Aeromonas hydrophila* invasion causing mortality in channel catfish. This could be the case in Kirinyaga County where the occurrence of parasites and bacteria of economic significance have been reported by Murugami et al. [31] and Wanja et al. [38]. Other infectious diseases of fish reported in central Kenya include infectious pancreatic necrosis [39] and infectious haematopoietic necrosis viruses [40] both from farmed rainbow trout and motile *Aeromonas septicaemia* [41].

#### 3.4.2. Parasitological Findings

Microscopic examination of gills, slime, and vitreous humor revealed numerous *Diplostomum* spp. (most prevalent), *Dactylogyrus* spp., and *Clinostomum* spp. (least prevalent) from fish from several farms that were reporting mortality at the time of sampling. *Diplostomum* spp. can occur free in the vitreous humor of the eyes of fish. In high numbers, they can impair vision, therefore causing blindness [9]. *Diplostomum* infested fish had extremely poor body condition.

**Figure 5:**
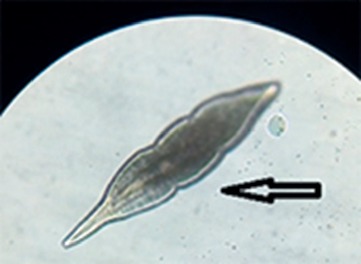
*Dactylogyrus* spp. (black arrow) isolated from gills of tilapia.

*Dactylogyrus* spp. are monogenean trematodes having one or two anteriorly placed eyespots and are found on the gills of their host (Figure 5). In intensive culture conditions, monogeneans are pathogenic to their hosts, especially young fish. In severe infections, they cause gill hyperplasia and skin erosions.

*Clinostomum* spp. are digenean trematodes which encyst on the muscle and skin causing a yellow appearance (yellow grub) that make the fish unsightly, thereby causing economic losses due to rejection.

**Figure 6:**
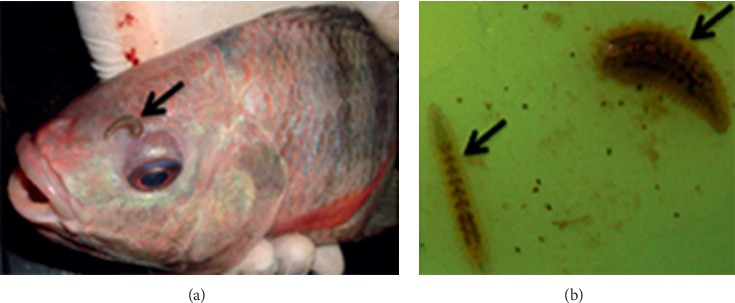
Nile tilapia (*O. niloticus*) showing a *Piscicola* leech above the eye (arrow in (a)). (b) Two detached leeches found in the source pond water.

*Piscicola* leeches were encountered in single tilapia only farm in an earthen pond (Figure 6). Macroscopically, affected fish had excess mucus. On necropsy examination, all the fish had pale friable livers. *Piscicola* spp. has been known to transmit fish trypanosomes and viruses such as infectious haematopoietic necrosis virus [9].

#### 3.4.3. Biosecurity Practices

**Figure 7:**
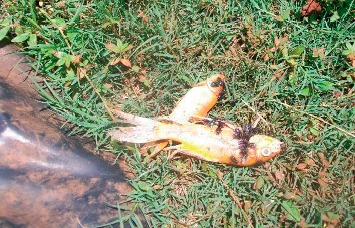
Dead goldfish disposed off near the pond.

Despite the significance of biosecurity, only 1% of the respondents reported to practice partial biosecurity measures including disinfection and traffic control. There are inadequate sanitary disposal arrangements for dead fish (Figure 7). In some farms, it was reported that dead fish would be left in ponds only to be feasted by other fish. Cannibalism of infected fish has been touted as possible routes of streptococcal transmission in fish [36].

Biosecurity aims at preventing transmission or spread of infectious diseases in an aquaculture facility. Most important diseases affecting farmed fish have been fortuitously carried to nonnative areas because of improper biosecurity measures [9]. Basic on-farm biosecurity principles include isolation of a new fish stocks before stocking within existing fish stocks/isolation of dead fish, sanitation, traffic control, water sterilization, and provision of safe feed [1].

### 3.5. Risk Factors for Fish Mortality/Disease

Case farms were those that experienced fish mortality and/or disease in the last six months. Association between experiencing fish mortality and potential risk factors was researched using univariate logistic regression [42]. The odds ratio for fish mortality was highest in farms that used livestock manure (1.500) while farms that experienced floods into the fishponds in the past one year had the lowest odds for fish mortality (Table 1).

Farms that used livestock manure for pond fertilization were 50% more likely to have experienced fish mortality than those that used chemical fertilizers. The wide use of livestock manure among fish farmers poses danger of transfer of disease-causing agents especially bacteria which may infect fish [43, 44]. Dry organic manure also consumes dissolved oxygen during the decomposition process.

The odds for mortality were 16.8% higher in farms where fishponds were overstocked compared to farms where fishponds were stocked optimally. Overcrowded culture systems lead to poor water quality since crowded ponds often have low dissolved oxygen levels, low pH values, and high nitrates and ammonia that increase daily after feeding [29]. Overstocking may result in atypical behaviour, e.g., cannibalism as a result of competition for feed. The skin is the primary barrier against pathogens; any break or injury or wounds on the skin enhances pathogen entry. In addition, competition for feed occasioned by high stocking density may lead to stunted growth and compromised immunity.

The odds for fish mortality were 12.9% higher in farms where fish were fed on homemade rations/livestock feeds and weeds compared to farms where fish were fed on properly constituted commercial feeds. Improper fish nutrition in the form of either underfeeding or feeding unbalanced diet or feeds of low-quality composition and digestibility leads to retarded growth and organ development as well as a weak immune system which predisposes fish to diseases. In addition, feeding excess protein-based feeds leads to high ammonia (waste product) levels in the water leading to poor water quality [45].

### 3.6. Assessment of Pond Water Suitability for Fish Farming

Water quality was assessed in 33 farms. The mean ± standard deviation values for physicochemical parameters of water samples taken from the studied ponds in different subcounties are presented in Table 2. Water temperature at the time of sampling was within the recommended limit and ranged from 20.5°C to 31.7°C.

Of the 33 fishpond water samples tested, 1 (3%) and 6 (18%) exceeded the recommended limits of <100 mg/L, <0.2 mg/L, and <3 mg/L of nitrate and nitrite, respectively. Of the 29 fishponds tested, 17 (59%) exceeded the recommended limits of 0.05 mg/L of total ammonia (NH_4_NH). The levels of dissolved oxygen were below desirable limits in 9% (*n* = 3) of the tested ponds. Six percent of the tested ponds had higher pH than the recommended limits of 8.5.

In this study, >50% of the ponds were found to be unfit for fish rearing based on their chemical parameters. Disease-causing agents are normally present in water and cause few problems; however, an imbalance due to water quality fluctuations and human activities may result in disease occurrence. Water quality is adversely affected by extrinsic factors originating outside the pond environment [30]. Some of these extrinsic factors relate to fish husbandry practices, e.g., excess organic materials such as manure. The findings of this study strongly suggest that poor water quality (e.g., high levels of ammonia) is a factor of consideration in the event of fish deaths. It is evident that probably most of fish kills are due to high levels of total ammonia. While studies have reported desirable limits for nitrite level [47,48], there are however no safer limits in any aquatic system. Poor water quality, e.g., elevated levels of nitrite or ammonia, has been associated with streptococcal infections in fish [36].

## 4. Conclusions

Fish diseases remain the most important factor affecting the growth and sustainability of aquaculture. Impacts of various management practices such as pond fertilization using raw livestock manure, high fish stocking density, and elevated levels of nitrates, nitrites, and high ammonia in fishponds require urgent address for long-term and successive aquaculture development. There is a need to strengthening disease control measures by ensuring optimal biosecurity measures and good husbandry practices (improved seining practices, proper nutrition, good water quality, and effective vaccination) to protect fish.

## Figures and Tables

**Table 1:** Odds ratio for fish mortality against some farm practices.

Risk factor	*χ* ^2^ (df = 1)	*P* < 0.05	Cramer's V	Odds ratio
Use of livestock manure for pond fertilization	0.227^a^	0.634	0.064	1.500
Overstocked fishponds	0.094^a^	0.759	0.034	1.168
Feeding fish on homemade ration (livestock feeds and weeds)	0.045^a^	0.832	0.024	1.128
Sharing fishing nets and other equipment with other farmers	0.026^a^	0.873	0.017	1.088
Occurrence of floods	0.001^a^	0.978	0.003	1.018

^a^Insignificant associations (*χ*^2^ (df = 1), *P* < 0.05).

**Table 2:** Mean ± SD values for physicochemical parameters of pond waters in different subcounties of Kirinyaga County.

Subcounties	Physicochemical parameter						
		Dissolved oxygen (mg/L)	Temperature (°C)	pH	Total ammonia (mg/L)	Nitrate (mg/L)	Nitrite (mg/L)
Kirinyaga Central	Mean ± SD	7.1 ± 2.8	25.9 ± 3.7	7.96 ± 0.7	0.06 ± 0.1	14.4 ± 10.5	0.07 ± 0.05
	Range	3.2–11.7^c^	21.6–31.7	7.35–9.2	0.0–0.2^c^	0.06–27	0.003–0.15

Kirinyaga West	Mean ± SD	5.8 ± 1.6	24.3 ± 1.1	7.4 ± 0.8	0.35 ± 0.8	43.0–31	0.15 ± 0.13
	Range	3.9–7.7^c^	23.3–26.0	6.7–8.6	0.0–1.7^c^	12.5–84	0.05–0.34^cc^

Mwea East	Mean ± SD	6.1 ± 2.0	28.0 ± 3.2	8.07 ± 0.8	0.93 ± 0.9	38.1 ± 50.1	0.12 ± 0.14
	Range	4.4–8.3^c^	24.5–30.8	7.2–8.5	0.29–1.6^cc^	12.9–113.2^c^	0.03–0.33^cc^

Kirinyaga East	Mean ± SD	6.2 ± 2.9	24.2 ± 2.4	7.46 ± 0.5	0.37 ± 0.6	22.4 ± 17.6	0.60 ± 1.08
	Range	2.3–13.40^c^	20.5–28.7	7–8.4	0–1.94^c^	0.0–49.2	0–3.06^c^

Mwea West	Mean ± SD	4.4 ± 3.9	26.2 ± 3.2	7.0 ± 0.6	2.98 ± 4.7	37.7 ± 15.4	0.10 ± 0.04
	Range	0.13–9.1^b^	22.7–30.9	6.27^a^–7.7	0.12–9.9^cc^	17.6–59.2	0.03^b^–0.14

County total	Mean ± SD	6.0 ± 2.8	25.3 ± 2.9	7.54 ± 0.7	0.72 ± 1.9	29.4 ± 24.5	0.27 ± 0.7
	Range	0.13–13.4^b^	20.5–31.7	6.27^a^–9.2	0–9.9^c^	0.0–113.2^c^	0.0–3.06^c^

The optimal ranges of water parameter for fish culture are as follows: dissolved oxygen = 1–5 mg/L; temperature = 20°C–32°C; pH = 6.5–8.5; total ammonia = 0–0.05 mg/L; nitrate = 0–100 mg/L; nitrite = 0.02–0.2 mg/L as described by Bhatnagar and Devi [46]. *Note*. ^cc^Both lower and upper values of the reported range exceeded the optimum range. ^a^Only lower value was below the optimum range. ^b^Lower value was below the optimum range and the upper value was above the optimum range. ^c^Only upper value was above the optimum range. ^*∗*^Significantly different (*P* < 0.001) across subcounties.

## Data Availability

Data associated with this article can be found, in the online version, at https://dx.doi.org/10.17632/y89spy3yfn.1#file-6a588b50-fab7-402f-9349-6c4c1bb8b500.

## Supplementary Materials

Supplementary Materials Questionnaire (submitted as a supplementary file) relied on evaluating fish farming practices in Kirinyaga.
