# Supplementary material for: Fish Husbandry Practices and Water Quality in Central Kenya: Potential Risk Factors for Fish Mortality and Infectious Diseases
Source: Vet Med Int. 2020 Mar 19;2020:6839354. doi: 10.1155/2020/6839354 (PMC7106927; doi:10.1155/2020/6839354)
Supplement: Supplementary Materials — Questionnaire (submitted as a supplementary file) relied on evaluating fish farming practices in Kirinyaga. [file 6839354.f1.pdf]

UNIVERSITY OF NAIROBI
COLLEGE OF AGRICULTURE AND VETERINARY SCIENCE
TRAHESA PROJECT

A Questionnaire for Fish Farmers on Assessment of Fish Farming Practices in Small-Scale Farms in Kirinyaga County, Kenya.
This questionnaire seeks your views on risk factors influencing water quality in fish farming. The information obtained is for research purpose and will be kept confidential.

Date of interview .... Telephone no. .... Code ....

Section A: Background Information

1. Sub-county_______________ Ward_________________Village _____________________
2. GPS readings:
   Eastings ___________ Northings/southings ____________ Elevation _____________
   Acreage of the farm ___________________
3. Type of farm house (*Please tick appropriately*)
   [1] Stone [2] Wooden [3] Earthen [4] Iron sheets [5] Others (Specify) ____________

Section B: Biodata

1. Name of owner _____________________________________
2. Age of owner?
   [1] 21-30 years [2] 31-40 years [3] 41-50 years
   [4] > 50years
3. Gender of the owner? [1] Male [2] Female
4. Main occupation of the owner:
   [1] Farming [2] Business [3] Salaried employee [4] Others (Specify) _________
5. Education level of the owner __________________
   [1] No formal education [2] Primary level [3] Secondary level
   [4] Tertiary level
6. Name of the respondent: ______________________________________
7. Relationship of respondent to owner: (*Please tick appropriately*)
   [1] Owner [2] Manager [3] Attendant [4] Family member [5] Others (Specify) ____________
8. Gender of respondent: [1] Male [2] Female
9. Education level of the respondent: (*Please tick appropriately*)
   [1] No formal education [2] Primary level [3] Secondary level
   [4] Tertiary level

Section C: Information on fish farm

1. Observe and note the type of ponds in the farm:

[1] Earthen [2] Liner ponds [3] Concrete
[4] Others (Specify) ___________

2. Observe and note the location of ponds?
[1] Residential areas [2] Rice fields [3] Along the valley [4] Others (Specify) _______________
3. Which fish species are cultured in the farm? (*Please tick appropriately*)
[1] Tilapia mixed sex [2] Tilapia mono sex [3] Catfish
[4] Tilapia-catfish poly culture [5] Ornamental [6] Others (Specify) _______________
4. What is the main reason for doing fish farming?
[1] Business [2] Subsistence [3] Hobby [4] Others (Specify)
5. Observe and note active crop farming [1] Absent [2] Present
6. Do you use commercial fertilizers for crop farming? [1] Yes [2] No
7. How often do you apply fertilizers to your crops (in a month)?

Section D: Farm size and stocking densities

1. How many ponds do you own? _____________
2. What is the size of each pond?(*Indicate the size and note stocking density/pond*)

**Pond**	**Size in**	**Stocking density**
[1] Pond 1		
[2] Pond 2		
[3] Pond 3		
[4] Pond 4		
[5] Pond 5		

3. Where did you source the first stock of fingerlings?

**Source of fingerlings (*Please tick appropriately*)**	**Please specify**
[1] Government breeding farms	
[2] Private breeding farms	
[3] From own ponds	
[4] From other farmers	
[5] Others	

4. When did you last restock your ponds (Year)?
5. Where does the farm source the restocking fingerlings?

**Source of fingerlings (*Please tick appropriately*)**	**Please specify**
[1] Government breeding farms	
[2] Private breeding farms	
[3] From own ponds	
[4] From other farmers	
[5] Others	

6. What were the previous stocking and harvesting numbers for the last 3 cycles? (*Starting with the most recent*) (Please fill in as *appropriate*)

**Cycle**	**Production cycle**	**Stocking numbers**	**Harvesting numbers**

1			
2			
3			

Section E: Water quality

1. What is the main source of water used in the pond? (*Please tick appropriately*)
   [1] River [2] Borehole [3] Dam [4] Harvested rain water [5]Others (Specify) _______________
2. Of the water sources in (1) above, which one is most suitable for fish rearing?
3. Why do you think it is most suitable?
   ____
4. Do you have rain water flowing through the ponds? If yes which months?
   ____
5. How frequently do you change water in the pond?
   [1] Twice per month [2] Once per month [3] Once every two months [4] Never [5] Others (Specify) ___________
6. What do you do to the pond before restocking? [1] Drain only [2]Drain and treat [3] Nothing
7. If the pond is drained, do you usually remove the bottom mud after harvesting?
   [1] Yes [2] No
8. Where do you normally drain/empty used water into?
   [1] Rice paddy [2] Other ponds [3] River [4] Agricultural land [5] Others (Specify) ____________
9. How is the pond treated?
   [1] Liming [2] Sun drying [3] both [4] Others (Specify) ____________
10. Have you ever conducted tests on your pond?
    [1] Yes [2] No
11. If yes, which parameters were measured?

**Parameters**	**Tick appropriately**
Dissolved oxygen	
Temperature	
Ammonia	
Nitrite	
pH	
Water colour	
Salinity	

12. How often do you check the parameters?
    [1] Everyday [2] Weekly [3] Monthly [4] In case of sick fish [5] Others(specify)
13. Is water availability a constraint to fish farming (*Rate appropriately)* [1] Major [2] Minor [3] Not a problem
14. Which months do you mainly experience water shortage __________________
15. Have you ever experienced flooding of your fish ponds in the last 1year?
    [1] Yes [2] No
16. If yes, which problems did it cause?

[1] Outbreak of diseases [2] Escape of fish from ponds [3] Introduced predators
[4] Others (Specify) ____________

Section F: Fish feeding

1. What type of fish feed do you use?
   [1] Rice/wheat bran and husk [2] Weeds, duckweed [3] No feed [4] Commercial fish feeds [5] Others (Specify)
2. How many times do you feed in a day?
   [1] Once [2] Twice [3] > Twice [4] Irregularly [5] Never [6] Others (Specify)
3. Is there difference in feeding regime between /among ponds?[1] Yes [2] No
4. If yes, which particular ponds and why?

Pond	Reason
[1] Pond 1	
[2] Pond 2	
[3] Pond 3	

5. Is feed availability a constraint to fish farming (*Rate appropriately*)[1] Minor [2] Major [3] Not a problem
6. If yes, in which months do you experience feed shortage?

Section G: Pond Fertilization

1. Is the pond fertilized before stocking fingerlings? [1] Yes [2] No
2. If yes, name the types of fertilizers used and give amounts in Kg?

**Type of fertilizer**	***Please specify name***	**Amount in Kg**
[1] Animal manure		
[2] Chemical fertilizer		
[3] Both		
[4] Others (specify)		

3. How frequently do you fertilize the pond? (In months)
   ____
4. In what form do you usually use manure?
   [1] Wet manure or slurry [2] Dry [3] Others (Specify) ___________
5. Do you practice integrated fish farming?
   [1] Yes [2] No
6. If yes, which animals? __________________________________

Section H: Fish Diseases

1. Are fish diseases a constraint to fish farming (Rate appropriately) [1] Minor [2] Major [3] Not a problem

2. How do you know that your fish are sick?
   [1] Response to feeding [2] Fish movement [3] Change in water color
   [4] Routine sampling and observe weight [5] Don't know [8] Others (Specify) ______
3. Have you observed a disease in your farm in the last 6months? ___________ (Month/Year).

4. Do you know the name of the disease? [1] Yes [2] No
5. If yes, what is the name? ___________

6. Did you observe any death when you had this problem?
   [1] Yes [2] No
7. If yes, how many fish died? [1] <10 [2] 10-20 [3] 21-30 [4] >30
8. What actions do you take when your fish are sick? (*Please tick appropriately*)
   [1] Call a Vet/ Paravet [2] Fish expert [3] Self-treat [4] Do nothing
   [5] Advice from other farmers [6] Others (Specify) ___________

9. Who harvests the fish in this farm?
   [1] Farm employees [2] Custom harvest crews [3] Fish processing harvest crews
   [4] Others (Specify) ___________
10. Do you share the fishing nets and other equipment with other farmers?
    [1]Yes [2] No
11. Do you use the same fishing net between ponds?
    I. [1]Yes [2] No
12. What do you do to the nets after harvesting?
    [1] Washing with water only [2] Washing and disinfecting [3] Drying in the sun
    [4] Never [5] Others (Specify) ___________
13. What challenges do you face as a fish farmer?
    I. **……………………………………………………………………..**
    II. **……………………………………………………………………..**
    III. **……………………………………………………………………..**
    IV. **……………………………………………………………………..**
14. Give possible solutions to these challenges
    I. ……………………………………………………………………….
    II. ………………………………………………………………………..
    III. ……………………………………………………………………….
    IV. ……………………………………………………………………….
    V. ……………………………………………………………………….

..........Thank you for taking your time to fill this questionnaire.................................

Water Quality Parameter Record Sheet

Pond number____________________

Pond		Water quality parameters					
		Temperature T^0c	pH	DO (mg/L)	Un-ionized NH_3 (mg/L)	Nitrite (mg/L)	Nitrate (mg/L)
A	**S_1**						
	**S_2**						
	**S_3**						
B	**S_1**						
	**S_2**						
	**S_3**						
